# Supplementary material for: Gamma Secretase Inhibitors as Potential Therapeutic Targets for Notch Signaling in Uterine Leiomyosarcoma
Source: Int J Mol Sci. 2022 May 26;23(11):5980. doi: 10.3390/ijms23115980 (PMC9180633; doi:10.3390/ijms23115980)
Supplement: Supplementary file 1 [file ijms-23-05980-s001.zip › ijms-1717485-supplementary.pdf]

Supplementary Materials

Table S1. Characterization of uterine tissue and uterine fibroid samples

Code	Pathology	Phase in the menstrual cycle
BH7	Uterine fibroids, adenomyosis	Proliferative
BH11	Uterine fibroids	Secretory
BH12	Uterine fibroids, adenomyosis	Proliferative
UF1	Uterine fibroids	Secretory
UF2	Uterine fibroids	Interval
UF3	Uterine fibroids, adenomyosis	Inactive

BH: benign hysterectomy; UF: uterine fibroid

Figure S1. Schematic layout of tissue array (TA) slide

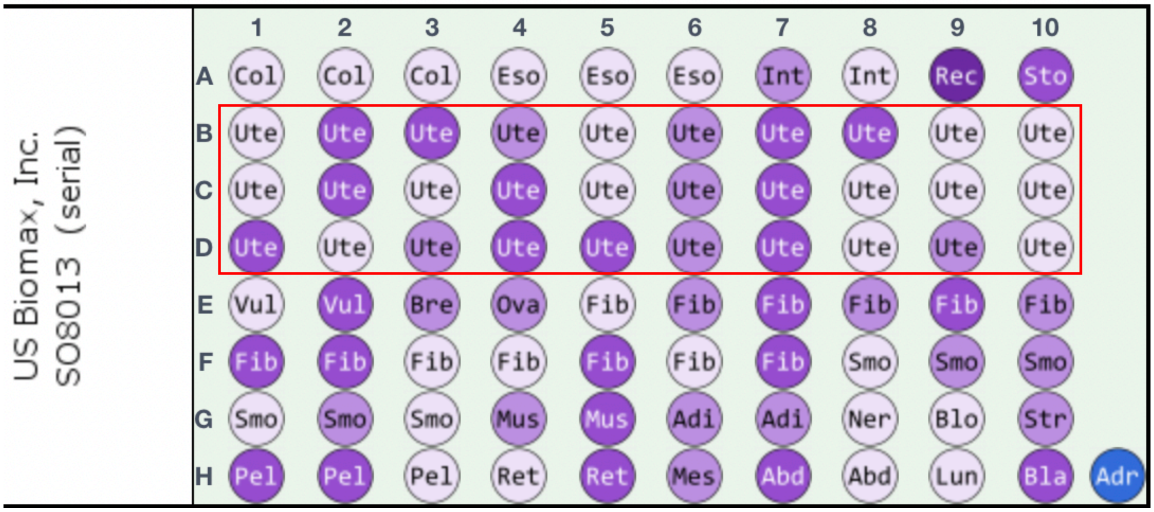

TA image provided by US Biomax, Inc. Thirty out of 80 samples are uterine leiomyosarcoma (uLMS) samples, outlined in red.

Table S2. Internal review of tissue array (TA)

**Score**	**Atypia**	**Necrosis**	**Mitotic Count**	**Hypercellularity**
None	4 (13%)	26 (87%)	14 (47%)	1 (3%)
Mild	12 (40%)	4 (13%)	11 (36%)	7 (24%)
Moderate	5 (17%)		2 (7%)	18 (60%)
Severe	9 (30%)		3 (10%)	4 13%)
total	30	30	30	30

Each uLMS sample is characterized by extent of atypia, necrosis, mitotic count, and hypercellularity, which are histologic features of uLMS. Frequency is reported.

Table S3. Antibodies for immunostaining

Protein	Company	Catalog Number	Dilution	Host
IgG	Abcam	ab172730	Varies	Rabbit
IgG	Abcam	ab37355	Varies	Mouse
Anti-NOTCH1	Cell Signaling	3608	1:100	Rabbit
Anti-NOTCH3	Abcam	ab23426	1:250	Rabbit
Anti-NOTCH4	Kitajewski Lab	RB2-2AFP	1:100	Rabbit
Anti-CD31	Novus Biologicals	NB100-2284	1:100	Rabbit
Anti-HES1	Abcam	ab119776	1:100	Mouse
Anti-KI-67	Abcam	ab15580	1:500	Rabbit
Anti-Rabbit, Alexa Fluor 594	Invitrogen	A21207	1:500	Donkey
Anti-Mouse, Alexa Fluor 555	Invitrogen	A21422	1:1000	Goat

Table S4. Primer sequences

**Gene**	**Forward**	**Reverse**
NOTCH1	5′-GCAGACTATGCCTGCAGCTG-3′	5′-GCCACACTCGTTGACATCCTG-3′
NOTCH2	5′-CAGTGTGCCACAGGTTTCACTG 3	5′-GCATATACAGCGGAAACCATTCAC-3′
NOTCH3	5′-CCTGTGGCCCTCATGGTATC-3	5′-CATGGGTTGGGGTCACAGTC-3′
NOTCH4	5′-ATGACCTGCTCAACGGCTTC-3′	5′-CATGGGTTGGGGTCACAGTC-3′
HES1	5′-AAAGATAGCTCGCGGCATTC-3′	5′-AGGTGCTTCACTGTCATTTCCA-3′
18S	5′-CCGGGCTTCTATTTTGTTGGT-3′	5′-TAGCGGCGCAATACGAATG-3′

Table S5. Antibodies for Western blot analysis

Protein	Company	Catalog Number	Dilution	Host
Anti-NOTCH1	Cell Signaling	3608	1:1000 in 5% skim milk	Rabbit
Anti-NOTCH2	Cell Signaling	4530	1:100 in 5% BSA	Rabbit
Anti-NOTCH3	Abcam	ab60087	1:1000 in 5% skim milk	Rabbit
Anti-NOTCH4	Cell Signaling	2423	1:1000 in 5% skim milk	Mouse
Anti-α-tubulin	Abcam	ab52866	1:2000 in 5% skim milk	Rabbit
Anti-Rabbit IgG (H+L) Cross Adsorbed Secondary Antibody, HRP	Invitrogen	G-21234	1:2000 in 5% skim milk	Goat
Anti-Mouse IgG (H+L) Cross Adsorbed Secondary Antibody, HRP	Invitrogen	G-21040	1:2000 in 5% skim milk	Goat

Figure S2. Proliferation and invasion assays of SK-UT-1B & SK-LMS-1

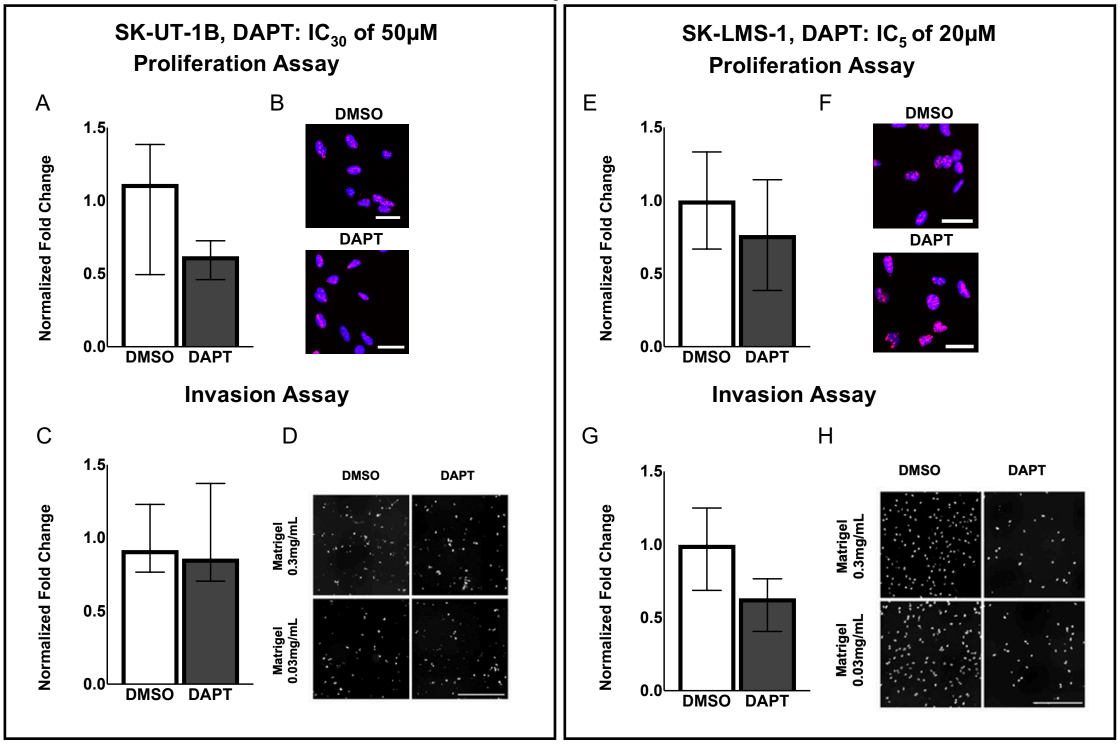

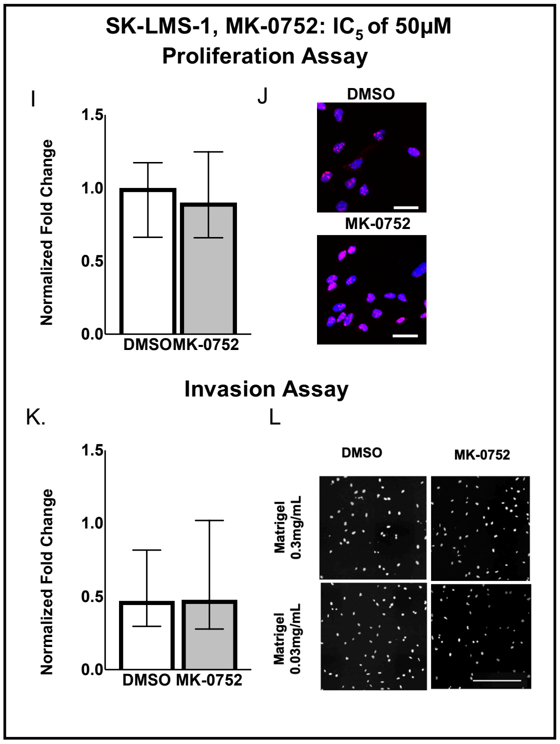

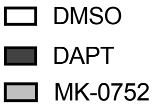

Figure S2. Proliferation and invasion assays of SK-UT-1B and SK-LMS-1. A-D, SK-UT-1B cells were exposed to DAPT at IC_{30} of 50 μM or DMSO control. Cellular proliferation and invasion were determined at 24 hours. Cellular proliferation **(A)** and invasion **(C)** were similar in cells exposed to DAPT and the DMSO control. Representative images of Ki-67 immunostaining **(B)** and SK-UT-1B cells after invasion through thick and thin Matrigel layers **(D)**. **E-L**, SK-LMS-1 cells were exposed to DAPT at IC_5 of 20μM or MK-0752 at IC_5 of 50μM or DMSO control. Cellular proliferation and invasion were determined at 24 hours. Cellular proliferation **(E, I)** or invasion **(G, K)** were similar after exposure to DAPT at IC_5 and DMSO control and after exposure to MK-0752 at IC_5 and DMSO control. **F, H, J, L,** Representative images of Ki-67 immunostaining and invasion assays. Data is presented as median + IQR. Scale bars=50μm.
